# Determinants of CO_2_ emissions generated by air travel vary across reasons for the trip

**DOI:** 10.1007/s11356-020-12219-4

**Published:** 2021-01-12

**Authors:** Martin Thomas Falk, Eva Hagsten

**Affiliations:** 1 School of Business, Department of Business and IT, University of South-Eastern Norway, Campus Bø; Gullbringvegen 36, 3800 Bø, Norway; 2 School of Social Sciences, University of Iceland, Reykjavík, Iceland

**Keywords:** Air travel CO_2_ emissions, Tourist air travel, Business air travel, Count data models

## Abstract

This study estimates factors of importance for the carbon dioxide equivalent (CO_2_e) emissions generated by travellers flying for different reasons based on representative Austrian micro data for the period 2014–2016. The annual average number of flights taken by adults vary between 0.1 (visiting friends) and 0.8 (going on holiday), and the amount of CO_2_e emissions generated by each return flight is approximately 1100 kg. This leads to a total of 6 million tonnes CO_2_e emissions per year. Results of the Pseudo Poisson Maximum Likelihood estimations reveal that the amount of CO_2_e emissions created is related to socio-demographic, locational and seasonal factors, although mainly for the largest group of travellers: the holiday makers. In this group, individuals with university degrees, young persons (16–24 years) and capital city residents generate the largest amounts of emissions, as opposed to persons with children and large households. Residents of the capital region each quarter cause 64 kg more CO_2_e emissions than inhabitants of rural areas, persons with university degrees create 74 kg larger emissions than those without degrees and young adults instigate 90 kg more emissions than middle-aged persons. CO_2_e emissions of holiday flights are highest in the first quarter of the year. The importance of education is also pronounced for CO_2_e emissions related to business travel, as is gender.

## Introduction

Services related to tourism, including air transportation, are increasingly questioned because of their presumptive negative impact on global carbon emissions. Air travel is considered to be the most environmentally damaging form of transportation with respect to climate change (Gössling and Upham 2009; Gössling and Humpe 2020) and emissions from aviation are more harmful than those from ground traffic (Lee et al. 2009). Findings based on research undertaken before the Covid-19 pandemic grounded most aircraft fleets in early 2020 point to the fact that a small group of individuals contributes to a large amount of the Co_2_ emissions (Gössling et al. 2009; Brand and Preston 2010; Gössling and Humpe 2020). Despite this, the characteristics of those individuals who generate the largest amount of flight-related emissions by reason for travel (holiday, visiting friends and relatives or business) are presently unknown.

The aim of this study is to gain more insights into aspects of importance for the CO_2_ emissions generated by air travellers with different reasons for their trips. Socio-demographic, locational and seasonal factors are employed to explain the amount of emissions at the individual level. The analysis uses a representative micro data set of 17,400 observations on Austrian residents that travel at least once per quarter for reasons of businesses, holidaying or visiting friends and relatives during the period 2014–2016. The Pseudo Poisson Maximum Likelihood estimator (PPML) is employed to estimate the relationships.

Until 2020, long-haul air travel is the fastest growing segment of passenger mobility (Airbus 2018) and ICAO (2009) estimates a 300% increase in emissions from air travel by 2050. Total aviation emissions are considered to account for 20% of the global tourism carbon footprints (Lenzen et al. 2018), while aviation itself represents between 2.0 and 2.5% of total annual CO_2_ emissions (Lee et al. 2009, Graver, Zhang and Rutherford, 2019). Current discussions encompass not only the sustainability of frequent flying, primarily by business travellers (Young et al. 2014), but increasingly also “unnecessary” leisure and holiday travel (Becken 2002; Holden and Norland 2005; Graham and Metz 2017).

With the deregulation of the European and other aviation markets and the subsequent emergence of low-cost airlines, the share of holiday air travel in total number of passengers is increasing (O’Connell and Williams 2005; Tsui 2017; Álvarez-Díaz et al. 2019). Recently, new groups of environmental activists have appeared that emphasise the emissions caused by flying, introducing the Swedish term “flygskam” (flight shame) (Gössling et al. 2019; Gössling 2019; Gössling et al. 2020).^1^ These groups advocate alternative transportation modes such as train, even if the travel time is ten or twenty folded.

Air travel for purposes of business, migration and education as well as to visit friends and relatives may be difficult to avoid. Many firms, institutions and organisations are active on the international arena and long-distance relationships are not uncommon. There are also national or European parliamentarians, for instance, who are expected to have a close relationship with their constituencies even if the distances are far. Yet, holiday travel by air could be prevented to a certain extent, as environmentally friendly means of transportation are available for short- or medium-long distances. In general, the distribution of emissions caused by air transportation is highly uneven, with few people accounting for the largest proportion (Brand and Preston 2010).

Hardly any studies examine the link between aviation emissions and socio-demographic characteristics. An exception is Bruderer Enzler (2017), who uses a two-part model to investigate the determinants of air traffic emissions based on the Swiss environmental survey. Other studies focus on greenhouse gas emissions of all individual travellers, independent of characteristics (e.g. Brand and Preston 2010), long-distance travellers (Reichert et al. 2016) or travel emissions generated by the urban population (Czepkiewicz et al. 2018a, 2018b; Czepkiewicz et al. 2019).

Another strand of the literature examines flying behaviour in general, independently of the amount of CO_2_ emissions caused. Examples of this include the behaviour of (a) the urban population in Iceland (Czepkiewicz et al. 2020), (b) international celebrities (Gössling 2019), (c) German holiday makers (Gössling et al. 2017) and (d) Swiss inhabitants and distances of their flights (Schubert et al. 2020). Dargay and Clark (2012) explore the determinants of travel for five different purposes (business, commuting, leisure, holidays and visits from friends and relatives), but without accounting for the emissions generated.

This study contributes results on a far more detailed level of air travellers and their carbon footprints than hitherto available based on a regularly re-occurring representative official survey. In addition, the analysis takes into account how the importance of socio-demographic, locational and seasonal factors varies across reasons for travel.

The paper is structured as follows: the “Conceptual background” section introduces the theoretical background and provides the main hypothesis, the “Empirical model” section presents the empirical approach while the “Data and descriptive statistics” section describes the dataset. The empirical results are revealed in the “Empirical results” section and the “Conclusion” section concludes.

## Conceptual background

Few studies investigate the characteristics of the group of air travellers that generate the largest amounts of CO_2_ emissions. A review of 27 studies examining the behaviour of long-haul travellers only encompasses three studies relating to CO_2_ emissions (Czepkiewicz et al. 2018a). Graham and Metz (2017) propose a distinction between “discretionary” leisure travel (including holiday travel) and “non-discretionary” business travel where air travels motivated by visiting friends and relatives are in principle voluntary but often indispensable.

Socio-demographic characteristics as well as location may have an important influence on CO_2_ emissions of individuals and households in general (Qu et al. 2016; Bülbül et al. 2020). Analyses of CO_2_ emissions associated with air travel reveal that socio-demographic characteristics and location of individuals are equally important (Reichert et al. 2016; Bruderer Enzler 2017; Czepkiewicz et al. 2018a; Czepkiewicz et al. 2018a, 2018b). Common features considered in these cases are age, gender, household type, education, occupation and income. Persons living in urban regions with airports close by are not only more likely to go by plane but are also using this opportunity regularly and subsequently generate more CO_2_ emissions (Czepkiewicz et al. 2018a). One reason behind this pattern is the so-called escape travel or compensation hypothesis (Heinonen et al. 2013; Reichert et al. 2016) postulating that high urban density limits the quality of life and thus creates demand for frequent weekend trips and other short breaks. Czepkiewicz et al. (2018a) mention that the positive relationship between urban density and long-distance travel behaviour is still significant when demographic and socio-economic variables are controlled for.

Research that explicitly models the CO_2_ emissions demonstrates that greenhouse gas emissions by urban residents are five times higher than those generated by individuals living in rural areas (Heinonen et al. 2013). Additionally, there are a number of studies that point to the importance of education and income as drivers of air travel emissions (e.g. Bruderer Enzler 2017). Likewise, the phase of life appears to be important for the emissions created. Based on the Swiss environmental survey, Bruderer Enzler (2017) finds that household characteristics and family size are important, while the role of gender is less obvious. Brand and Preston (2010), for instance, suggest that gender is not significantly related to overall emissions from private, non-business travel while Brand and Boardman (2008) show that single-person households produce the highest average travel emissions per person, mainly caused by air travel.

Unfortunately, recent literature is difficult to compare because of variations in sample sizes, definitions and calculations of CO_2_ emissions from air travel (total air travel emissions or by purpose) as well as estimation methods used (multivariate or bivariate). There are, however, a few common denominators indicating that socio-demographic factors are of importance, although possible differences between leisure (holiday and visiting friends or relatives) and business travellers are largely neglected. Influenced by the determinants highlighted in the literature, and the gaps revealed, the emissions generated are analysed for the travel purpose (holiday, business, visiting friends or relatives) together with socio-demographic, location and seasonal characteristics based on a representative sample of trips and travellers. Data available on destination country makes it possible to calculate the amount of emissions as carbon dioxide equivalents (CO_2_e) caused by the flights. This leads to the main hypothesis (H1):

*H1: The determinants of CO*_*2*_*e emissions generated by air travel vary across reasons for the trip.* Implicitly, the hypothesis rests on the assumption that there is a relationship between emissions generated and socio-demographic, locational and seasonal factors.

## Empirical model

There are numerous studies on the choice to travel by air (Czepkiewicz et al. 2018a). This study follows Bruderer Enzler (2017) and Reichert et al. (2016), who model the amount of air travel–related CO_2_e emissions, *CO*2*e*_*itp*_, as a function of several socio-demographic factors including location, departure quarter and departure year:

$$ {\displaystyle \begin{array}{c} CO2{e}_{it p}={\beta}_{0p}+\sum \limits_{A=1}^5{\beta}_{jpA} AGECA{T}_{it}^A+\sum \limits_{E=1}^2{\beta}_{jpE} ED{U}_{it}^E+{\beta}_{jpW}{WOMEN}_{it}\\ {}+{\beta}_{jpC} CHILDRE{N}_{it}+\sum \limits_{S=1}^3{\beta}_{jpL}{LABOURSTATUS}_{it}^L+\sum \limits_{H=1}^5{\beta}_{jpH}{HHSIZE}_{it}^H\\ {}+\sum \limits_{F=1}^8{\beta}_{jpF}{FEDSTATE}_{it}^F+\sum \limits_{Y=1}^2{\beta}_{jpY}{YEAR}_{it}^Y+\sum \limits_{Q=1}^3{\beta}_{jpQ}{QUARTER}_{it}^Q+{\varepsilon}_{ipt},\end{array}} $$

where *i* is the individual, *t* denotes quarterly data (2014:1 to 2016:4) and *p* is reason for travel (holiday, visit friends and relatives or business). The explanatory variables encompass *AGECAT* denoting age class, *EDU* reflecting the level of education with no degree as the reference category and *WOMEN* if the traveller is female. *CHILDREN* is a dummy variable for travelling at least once with children, *HHSIZE* is a set of dummy variables measuring the household size with one reflecting the reference category and *LABOURSTATUS* is a group of dummy variables for the labour market position (employed, unemployed, student or retired, with unemployed as reference category). Variable *FEDSTATE* relates to the region where the traveller resides with the province Lower Austria as the reference category. Macroeconomic factors such as price effects and fluctuations of the business cycle are captured by annual year dummy variables *YEAR, QUARTER* controls for calendar effects within a single year with the first quarter (January to March) as the reference category and *ε*_*ipt*_ is the error term.

The Pseudo Poisson Maximum Likelihood estimator can be used to assess the determinants of the CO_2_e emissions generated by different groups of travellers. Santos Silva and Tenreyro (2006) argue that this estimator is suitable for dependent variables that contain a large proportion of zero values as in this case, where 82% of the leisure travellers and 95% of the business travellers did not fly at all in a given quarter and thus generate no CO_2_e emissions. Another advantage is that the PPML estimator is consistent in the presence of heteroscedasticity. The CO_2_e emission equation can be written in its exponential form (subscript *t* omitted):

$$ {CO}_2{e}_i=\mathit{\exp}\left({X}_i\ss \right)+{\epsilon}_i, $$

where *X* is the vector of explanatory variables mentioned above (all in form of dummy variables) and *ß* contains the parameters to be estimated.

## Data and descriptive statistics

Data for this analysis originate from the official Austrian travel survey (Statistics Austria 2017). This is a quarterly representative survey on holiday and business travels with at least one overnight stay, undertaken by persons living in Austria aged 15 years or older. The survey is stratified by federal state, age and gender. Each quarter, around 3500 randomly selected persons are interviewed by telephone. Participation in the survey is voluntary. The dataset encompasses information on actual domestic as well as international (outbound) trips by destination and purpose, length of stay, accommodation type, departure month, transportation mode and expenditures. A wide range of socio-demographic factors accompany the data such as educational attainment, gender, age class, labour market status, travel company size and federal state where the traveller resides. Although information is available from 2012 onwards, methodological changes of the travel survey restrict the estimation sample to the period 2014–2016.

Data on destination country makes it possible to calculate the amount of CO_2_ emissions (expressed in carbon dioxide equivalents) caused by the flights. The largest destination airport in each country is used for this exercise. There are two different emission calculators available (https://co2.myclimate.org/en/flight_calculators/new and https://www.icao.int/environmental-protection/Carbonoffset/Pages/default.aspx) (Table 4, Appendix) although the one from the ICAO has a limited coverage of airports and is thus not used here. The myclimate flight calculator determines the amount of CO_2_ emissions that an aircraft generates per passenger for a given flight route using the real distance. Nitrogen compounds and aerosols are also taken into account and converted into CO_2_. Business flights are associated with 30% more CO_2_ emissions for short- and medium-haul flights. Since information is not available on the number of flights in business class, the calculation method for economy flights is used for all flights as in Reichert et al. (2018). CO_2_e emissions generated by each quarterly trip are aggregated to the individual level. Although Baumeister (2017) concludes that almost no single flight generates similar emissions to another, depending on the number of stops and the vintage of the plane, a more detailed calculation of the emissions cannot be made here because information about the travel itself is not available.

Descriptive statistics also reveal that 1% of the holiday travellers and 1% of the business air travellers account for one-fifth and almost two-thirds of CO_2_e emissions during the sample period in their respective groups (Table 1). Emissions generated by flights to friends and relatives are negligible in this context.

**Table 1:** Amount of CO_2_e emissions generated by different groups of air travellers (kg)

	Holiday	Visit friends or relatives	Business
	Total		
2014	1,040,046	131,970	382,938
2015	1,075,104	141,004	327,740
2016	1,086,714	206,876	340,358
	Upper one percentile of emitters		
2014	199,718	89,174	250,866
2015	226,692	101,876	208,306
2016	218,786	155,320	214,724
	Contribution of the upper one percentile, per cent		
2014	19.2	67.6	65.5
2015	21.1	72.3	63.6
2016	20.1	75.1	63.1

Source: Austrian travel survey and https://co2.myclimate.org/en/flight_calculators/new

Based on the average number of holiday flights per person and year (0.8), the amount of CO_2_e emissions can be calculated. The emissions are then scaled up to the total adult population of 7.6 million in Austria, of which 60% goes on holiday (European Comission 2016). With the corresponding CO_2_e emissions of 1100 kg per person and flight, this results in a total amount of CO_2_e emission per year of approximately 6 million tonnes from air travel (and 4.0 million tonnes from the holiday travel). Based on a representative travel survey for Sweden, Åkerman (2012) calculates 4.2 million tonnes of CO_2_ equivalent emissions from international air travel for the period 2015–2016 (1 year, including all types of flights). In addition, descriptive statistics show that the CO_2_e emissions per person with at least one flight vary markedly over quarters, where the highest appears in the first quarter, reflecting the longer flight distances during this time of year (Table 2).

**Table 2:** Average number of flights per quarter and CO_2_e emissions per flight in 2014–2016

	Number of flights		
	Holiday	Visit friends or relatives	Business
2014 Q1	0.16	0.03	0.09
2014 Q2	0.21	0.03	0.09
2014 Q3	0.27	0.02	0.06
2014 Q4	0.17	0.03	0.09
2015 Q1	0.20	0.04	0.08
2015 Q2	0.21	0.03	0.09
2015 Q3	0.23	0.02	0.05
2015 Q4	0.14	0.04	0.08
2016 Q1	0.14	0.03	0.07
2016 Q2	0.21	0.03	0.09
2016 Q3	0.23	0.03	0.05
2016 Q4	0.15	0.05	0.08
Sum 2014 Q1–Q4	0.81	0.12	0.34
Sum 2015 Q1–Q4	0.78	0.12	0.30
Sum 2016 Q1–Q4	0.74	0.15	0.28
	CO_2_e emissions per person in kg (persons with at least one flight in any of the categories)		
	Holiday	Visit friends or relatives	Business
2014 Q1	1534	831	1251
2014 Q2	820	851	1228
2014 Q3	817	758	1276
2014 Q4	1091	991	1241
2015 Q1	1364	890	1205
2015 Q2	914	816	1096
2015 Q3	835	1007	992
2015 Q4	1236	1111	1275
2016 Q1	1670	1059	1233
2016 Q2	742	1105	859
2016 Q3	876	876	1185
2016 Q4	1257	1027	1654
Mean 2014 Q1–Q4	1065	858	1249
Mean 2015 Q1–Q4	1087	956	1142
Mean 2016 Q1–Q4	1136	1017	1233

Source: Austrian travel survey. Average Co2 emissions per flight in kg are calculated based on a return flight using https://co2.myclimate.org/en/flight_calculators/new

**Fig. 1:**
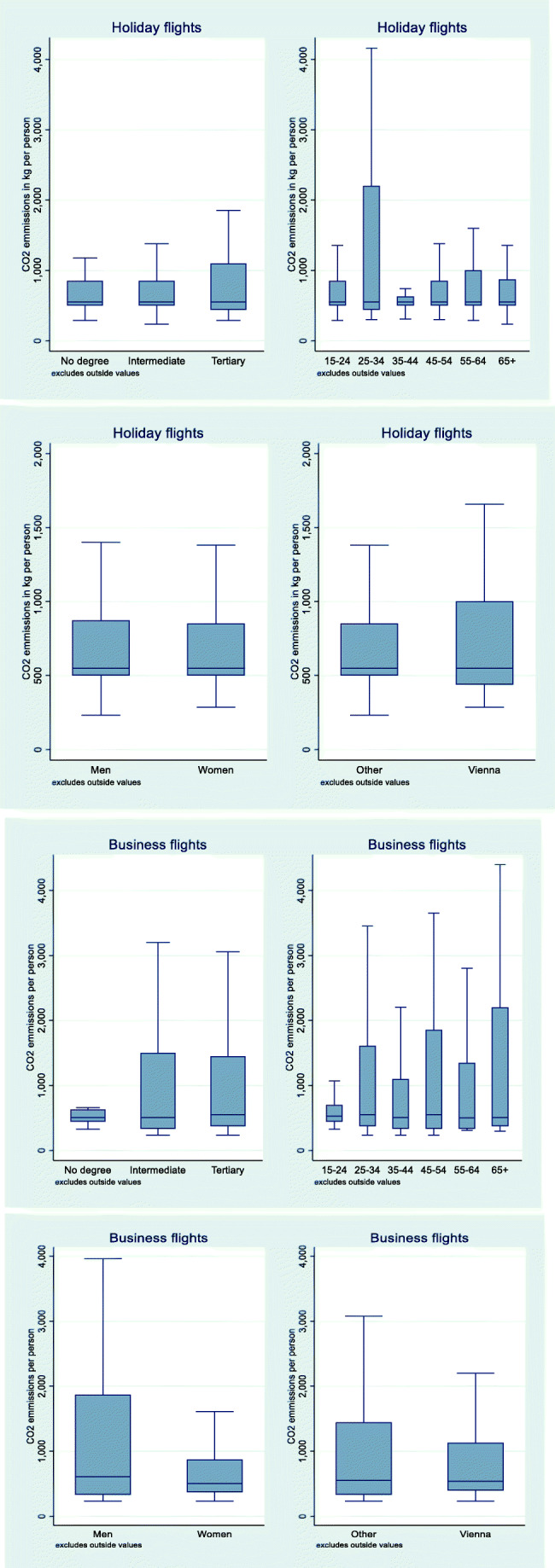
Box plots of individual CO_2_e emissions per group of travellers. Source: Austrian travel survey

The CO_2_e emissions generated by each holiday traveller (with at least one flight per quarter) are larger for highly skilled individuals (tertiary degrees), those living in the capital city of Vienna and for young people (Fig. 1).

Among business air travellers, those with higher education account for the majority of emissions (Table 5, Appendix). CO_2_e emissions created are higher in the older age classes of holiday travellers.

## Empirical results

The Pseudo Poisson Maximum Likelihood estimations show that the amount of COe_2_ emissions generated by air travellers residing in Austria relates to socio-demographic, locational and seasonal factors, although mainly for the largest group of travellers: the holiday makers (Table 3). In this group, young adults, those with tertiary degrees, residents of the capital city and men leave larger traces of CO_2_e emissions. Individuals travelling with children and those in large household generate far less emissions. There is also a strong seasonal pattern, where the lowest CO_2_e emissions can be observed for the second and fourth quarters of the year.

**Table 3:** PPML estimations of the amount of CO_2_e emissions generated by air travel 2014–2016

		(i)			(ii)			(iii)	
	Holiday			Visit friends and relatives			Business		
	dy/dx		z-stat	dy/dx		z-stat	dy/dx		z-stat
Age 15–24 (ref. cat. 35–44)	89.66	***	4.05	14.45	*	1.97	15.81		0.95
Age 25–34	55.53	***	3.05	5.71		0.85	6.37		0.65
Age 45–54	48.31	***	2.97	− 5.38		− 0.78	4.02		0.43
Age 55–64	23.41		1.25	2.46		0.32	− 3.19		− 0.26
Age 65+	29.53		1.22	− 5.21		− 0.50	− 9.72		− 0.47
Education medium (ref. low)	22.23		1.53	− 1.39		− 0.21	5.87		0.46
Education tertiary level	74.00	***	4.42	15.95	**	2.29	76.05	***	5.57
Women	19.91	**	2.24	6.45	*	1.72	− 68.50	***	− 8.31
Travellers with children	− 65.62	***	− 4.43	− 4.48		− 0.84			
Employed (ref. unemployed)	7.80		0.26	5.73		0.56	52.00	***	2.65
Student	2.97		0.09	19.01		1.63	42.18	*	1.89
Pensioners/out of labour force	− 0.95		− 0.03	18.26		1.48	− 42.52	*	− 1.65
Burgenland (ref. Lower Austria)	− 22.74		− 0.76	8.10		0.50	− 35.96		− 1.40
Vienna	63.77	***	4.79	24.37	***	4.22	24.16	**	2.33
Carinthia	− 61.06	**	− 2.49	− 6.42		− 0.71	− 3.05		− 0.17
Styria	− 30.07	*	− 1.80	− 10.73		− 1.37	− 5.33		− 0.46
Upper Austria	− 25.74	*	− 1.78	− 1.52		− 0.20	− 11.76		− 1.08
Salzburg	− 3.42		− 0.17	16.61	**	1.96	− 7.92		− 0.46
Tyrol	− 6.59		− 0.34	10.92		1.35	− 36.68	**	− 2.11
Vorarlberg	− 0.48		− 0.02	16.66	*	1.90	31.69	**	2.10
Household size = 2 (ref. = 1)	27.19	*	1.85	− 11.66	**	− 2.12	− 19.64	*	− 1.82
Household size = 3	− 17.92		− 1.07	− 7.59		− 1.25	− 25.27	**	− 2.29
Household size = 4	− 41.45	**	− 2.30	− 14.00	**	− 2.03	− 22.74	**	− 2.05
Household size = 5	− 52.28	**	− 2.30	− 14.68	*	− 1.74	− 11.51		− 0.95
Household size = 6	− 71.55	**	− 2.47	− 2.30		− 0.25	− 21.50		− 1.00
Year 2015 (ref. year 2014)	− 6.45		− 0.60	0.07		0.01	− 14.57	*	− 1.82
Year 2016	− 14.75		− 1.37	9.18	**	2.06	− 14.93	*	− 1.85
Quarter 2 (ref. quarter 1)	− 65.25	***	− 4.94	− 1.79		− 0.34	2.19		0.25
Quarter 3	− 30.81	**	− 2.56	− 7.74		− 1.48	− 19.25	**	− 2.11
Quarter 4	− 65.40	***	− 4.46	8.21		1.55	8.59		0.85
Number of observations	17,374			17,374			17,374		
Log pseudolikelihood	− 6,390,698			− 1,774,704			− 3,087,037		

Notes: Asterisks ***, ** and * denote significance at the 1, 5 and 10% levels. dy/dx denotes the marginal effects. Estimated by the Poisson pseudo-maximum likelihood estimator. Source: Austrian travel survey, Statistics Austria

The labour market status is not related to the amount of CO_2_e emissions created by holiday travellers. CO_2_e emissions resulting from flights to friends and relatives show that the main aspects of importance are the educational level and the capital region, while the remaining factors are of less or no importance. Since the capitals attract highly educated individuals, it can be expected that there is also a larger amount of residents with families elsewhere. Emissions related to business travels are crucially related to the educational level, labour market status, gender and location. Season is far less important, but there is a reduction in the summer quarter (July to September).

The marginal effects (dy/dx) of the PPML estimations directly indicate the strength of the associations and reveal that young holiday travellers aged 15–24 years produce the highest amount of CO_2_e emissions per quarter compared to the reference category 35–44 years (90 kg more). Persons with higher education generate 74 kg and 76 kg more emissions for holiday and business travels, respectively, than those without degrees. Inhabitants of the capital region are responsible for an addition of 64 kg emissions for holiday flights than people living in the rural provinces. Strong associations can also be observed for holiday travellers with children (− 66 kg) and persons living in large households (− 52 kg and − 72 kg in households with 5 and 6 or more persons, respectively). Emissions from holiday flights are lowest in the spring and autumn months (− 65 kg each). In terms of emissions from business flights, women relate to a reduced amount of emissions with 68 kg. Overall, the results mean that the hypothesis formulated cannot be rejected.

In general, the results do not deviate from the recent but fragmented literature, although the analysis performed here goes beyond earlier research both with respect to the large representative dataset and the calculation and modelling of flight-related CO_2_e emissions by travel purpose. Estimates based on total emissions mask the heterogeneity of the air travel behaviour among different groups, where emissions created by those travelling to visit friends and relatives are less related to socio-demographic factor than for the holiday makers. This approach also allows a ranking of the importance of the explanatory variables, where young persons, those with higher degrees or residents of the capital city generate more CO_2_e emissions for their holiday flights and highly educated persons and men for those of business flights.

Several robustness checks have been conducted. First, other CO_2_e calculation methods are used. The findings are not sensitive to the choice of the CO_2_e calculator (results are available upon request). Second, the central variables age, education and place of residence are interacted to investigate possible moderating effects. The results for holiday flights show that persons with tertiary education living in the capital city cause the highest amount of emissions (unreported results are available upon request).

Given that the emissions generated clearly vary across kind of travellers, with the holiday makers being responsible for the largest amount, possible policy interventions need to be customised. Literature suggests both voluntary initiatives and soft measures as well as hard actions (flight taxes, emissions taxes, carbon budget) to reduce the emissions of holiday flights (Becken 2007; Higham et al. 2016; Shaari et al. 2020), with hard measures being considered the most effective ones (Higham et al. 2016). Gössling et al. (2020) show that a two-thirds majority of survey respondents are in favour of market-based measures that increase the cost of flying, policies that force airlines to reduce their emissions and legislation to abolish subsidies. Using a willingness-to-pay approach, Seetaram et al. (2018) demonstrate that travellers are willing to pay a higher departure tax for business class and long-haul travel. Another policy option is to replace short-haul and domestic flights with train connections (Dällenbach 2020). According to Baumeister (2019), airplanes have no advantage over trains for distances under 400 km and the emission reduction potential would be particularly pronounced if the trains were run with renewable energy, shifting the responsibility of cleaner aviation to the supply side, where new technologies might be used as alternative solutions. Several airlines experiment with biofuels, for instance, with mixed evidence (Filimonau et al. 2018; Lu 2018; Efthymiou and Papatheodorou 2019). Other options suggested are electric airplanes, but this is not for the near future (Baumeister et al. 2020).

## Conclusions

This study provides novel empirical evidence on aspects of importance for the carbon dioxide equivalent (CO_2_e) emissions caused by different groups of air travellers, based on a large representative dataset on travel behaviour by Austrian residents for the period 2014–2016. Poisson Pseudo-Maximum Likelihood estimations show that the amount of CO_2_e emissions generated by different groups of travellers depend on socio-demographic, locational and seasonal factors, although mainly so for the largest group of travellers: the holiday makers. Education, location of residence, age and season are aspects most relevant for CO_2_e emissions generated by this group of travellers while education and gender (men) are driving CO_2_e emissions by business travellers. Socio-demographic, locational and seasonal factors are of less or no importance for emissions related to visiting friends and relatives.

The results imply that presumptive policy measures to reduce travel by air need to be customised. Given that the largest amount of emissions are produced by persons with higher degrees, supposedly not sensitive to air fares, additional measures targeting the demand side such as flight taxes might not be effective in reducing emissions. Instead, focus might need to shift to the supply side and to new technologies.

Travel surveys from the national statistical office are commendable sources for the analysis of the CO_2_e emissions and are generally available in a large group of countries. Future studies should be based on comparable data for a larger group of countries.

## Data Availability

Official data underlying the study can be ordered from Statistics Austria: http://www.statistik.at/web_de/services/mikrodaten_fuer_forschung_und_lehre/datenangebot/standardisierte_datensaetze_sds/index.html#index18

## Appendix

**Table 4:** List of airports and CO_2_ equivalent emissions for return flights starting in Vienna

Country	Airport code	Passenger CO_2_e/pax/return (KG)	
		IACO	co2.myclimate.org
Belgium	BRU	220.6	408
Denmark	CPH	197.0	396
Germany	FRA	153.4	328
Finland	HEL	281.2	550
France	CDG	223.2	438
Greece	ATH	247.4	502
United Kingdom	LHR	251.0	500
Ireland	DUB	310.0	628
Italy	FCO	195.2	370
Luxembourg	LUX	185.6	368
Netherlands	AMS	209.0	416
Portugal	LIS	392.2	832
Sweden	ARN	289.4	504
Spain	PMI	277.2	548
Iceland	KEF	454.4	1020
Norway	OSL	286.8	530
Switzerland	ZRH	156.0	322
Baltic States (Estonia, Latvia, Lithuania)	RIX	226.4	454
Croatia	SPU	127.8	298
Malta	MLA	263.4	526
Poland	WAW	166.8	308
Romania	OTP	200.4	382
Slovakia	KSC	n.a.	256
Slovenia	LJU	n.a.	232
Turkey	AYT	301.0	628
Czech Republic	PRG	107.8	236
Hungary	BUD	89.0	220
Cyprus	LCA	344.2	740
Bosnia and Herzegovina	SJJ	144.4	294
Serbia	BEG	122.0	286
Bulgaria	SOF	201.0	376
Russia	VKO	225.2	606
Rest of Europe	KBP	221.2	446
Egypt	CAI	372.6	852
Tunisia	TUN	277.6	522
Rest of Africa	CPT	1154.4	3000
USA	EWR	780.8	2200
Canada	YYZ	640.6	2200
Central and South America	GIG	n.a.	3200
China	PEK	659.6	2400
Rest of Asia	BKK	741.4	2800
Australia, New Zealand and islands north-east of them in the Indian Ocean	SYD	n.a.	5600

Note: Carbon dioxide equivalent (CO_2_e) emissions refer to a return flight in the economy class

Source: https://co2.myclimate.org/en/flight_calculators/new and https://www.icao.int/environmental-protection/Carbonoffset/Pages/default.aspx

**Table 5:** CO_2_ equivalent emissions by travel purpose and characteristics (2014–2016 per year on average)

	Holiday	Visit friends and relatives	Business
	Age class		
Age 15–24	149,987	31,637	42,061
Age 25–34	147,586	23,303	69,492
Age 35–44	114,967	19,657	71,300
Age 45–54	232,321	21,889	93,893
Age 55–64	209,535	32,777	54,158
Age 65+	212,515	30,688	19,442
	Education		
No degree	140,241	25,908	28,804
Education medium (ref. low)	588,495	73,613	125,485
Education tertiary level (university)	334,310	60,220	194,853
	Children		
Travellers without children	953,099	139,703	
Travellers with children	113,812	20,247	
	Gender		
Men	479,351	65,081	258,291
Women	587,560	94,869	92,054
	Labour market status		
Employed	625,065	79,477	294,857
Unemployed	7131	436	865
Student	104,954	26,215	30,503
Pensioners/out of labour force	306,820	50,603	19,589
	Federal state		
Burgenland	24,463	3921	4349
Lower Austria	214,381	22,467	63,390
Vienna	298,087	61,015	118,511
Carinthia	43,749	5049	16,561
Styria	117,865	10,399	37,699
Upper Austria	176,731	20,924	52,360
Salzburg	68,081	13,519	17,789
Tyrol	76,701	13,214	13,829
Vorarlberg	46,853	9441	25,857
	Household size		
Household size = 1	131,148	29,400	57,703
Household size = 2	449,902	52,675	92,257
Household size = 3	192,613	30,444	67,095
Household size = 4	186,356	27,054	79,995
Household size = 5	70,164	11,089	37,861
Household size = 6	36,728	9287	15,434

Note: CO_2_ equivalent emissions. Source: Austrian travel survey

## Footnotes

1 See for instance article in Time Magazine, May 16, 2019 “Now I Am Speaking to the Whole World.’ How Teen Climate Activist Greta Thunberg Got Everyone to Listen” (https://time.com/collection-post/5584902/greta-thunberg-next-generation-leaders/).
